# Rehabilitation of the severely atrophied dentoalveolar ridge in the aesthetic region with corticocancellous grafts from the iliac crest and dental implants

**DOI:** 10.4317/medoral.21146

**Published:** 2016-07-31

**Authors:** Miikka Lehmijoki, Heli Holming, Hanna Thorén, Patricia Stoor

**Affiliations:** 1 DDS. Department of Oral and Maxillofacial Surgery, University of Helsinki, Helsinki Finland; 2 DDS. Department of Prosthodontics, Oral and Maxillofacial Diseases, Helsinki University Central Hospital, Finland; 3 DDS, MD, PhD. Department of Oral and Maxillofacial Surgery, University of Helsinki, Helsinki Finland; 4 DDS, MD, PhD. Department of Oral and Maxillofacial Surgery, Helsinki University Central Hospital, Finland

## Abstract

**Background:**

The aim of this study was to assess changes in bone volume after block bone augmentation and placement of dental implants and further evaluate the aesthetic outcome of the treatment.

**Material and Methods:**

9 Patients with atrophied anterior maxilla were included in this study. They received total of 21 implants. Dimensions of the alveolar ridge were measured from cone-beam computed tomography x-rays. The bone level at the implant sites was analysed from intraoral x-rays and the aesthetic outcome was assessed from clinical photographs using a pink aesthetic score (PES) scaling.

**Results:**

The mean gained horizontal bone width at the marginal crest and 5 mm apically was accordingly 2.7mm and 5.0 mm. The mean PES rating was 9.8/14. The survival rate of.

**Conclusions:**

Reconstruction of the atrophied anterior maxilla with bone blocks and dental implants is a safe procedure with high survival rate and acceptable aesthetic outcome.

**Key words:**Dental implants, aesthetic region, corticocancellous bone grafts, pink aesthetic score, survival rate.

## Introduction

Rehabilitation of the aesthetic region, the anterior maxilla, with dental implants presents a complex challenge in cases where the patient has a high smile line. With severe atrophy of the dentoalveolar ridge the outcome is even more vulnerable to poor aesthetic outcome. Severe atrophy of the dentoalveolar ridge may be due to long term edentulism, hypodontia, trauma or earlier surgery in the area due to infection, unerupted teeth, or tumours.

The dentoalveolar ridge can be augmented for ideal implant positioning by various grafting techniques and materials. Extraction socket augmentation at time of extraction to avoid loss of bundle bone is the ideal (1), but this is not possible in all cases. To gain lost horizontal dimension different augmentation techniques can be used. In cases where the needed augmentation is moderate, guided bone regeneration (GBR) at time of implant placement is the treatment of choice. Different grafting materials have been studied excessively for GBR including xenograftic, alloplastic and allograftic materials (2-4). GBR has the disadvantage of an increased resorption compared to augmentation with autogenous bone blocks (5) unless simultaneously using non-resorbable membranes, which needs to be removed before placing implants and have a tendency to perforate the mucosa and get infected (6). When using allografts and xenografts there is also always the potential risk of immunological reactions and spreading of infection (7). However, there are studies showing safety of bovine bone used in GBR (8).

In severe atrophy of the dentoalveolar crest, the ridge being less than 3mm wide, a safe treatment option is to augment the ridge before placing implants. In these cases augmentation with bone grafts can be used. Bone can be used either as block or chips (9). Autogenous bone has been the “golden standard” and is grafted mainly intraorally, but also from the iliac crest or calvarium. Bone augmentation with a transplant from the mandible can produce excellent results (10), however, the available bone volume for grafting is limited and the bone is mainly cortical bone. The iliac crest is a donor site with greater supply of bone and the harvesting comes with minor and well-tolerated morbidity (11). The quality of the bone of the iliac crest is superior to grafts of the mandible or calvarium being both cortical and cancellous, thus containing additional bone marrow originating stem cells and osteoprogenitor cells.

The outcome of implant therapy is usually measured by the survival rate of implants with special criteria of success described by Albrektsson (12). In cases where the aesthetic values are important these standards only are insufficient considering the treatment outcome. The anterior maxilla is a sensitive area when focusing not only on a functional but also on an aesthetic outcome. Especially demanding are patients with a high smile line, a regular surrounding gingival margin and a thin biotype combined with severe atrophy of the dentoalveolar ridge.

In year 2005 Furhauser and co-workers (13) proposed a new measurement scaling, the pink aesthetic scoring (PES), focusing on the soft tissue appearance surrounding the implant restorations. With this method it is possible to objectively analyse the aesthetic outcome of the area rehabilitated with dental implants and augmentation procedures. The PES scale has seven different variables including forms of papillas, soft-tissue level, soft-tissue contour, soft-tissue color, soft-tissue texture, and alveolar process deficiency and it is considered to be an accurate tool for evaluation of aesthetic success of implants placed in anterior maxilla (14). According to previous studies the threshold for clinical acceptability is defined as a PES ≥ 8 (15) or PES over 6 (16).

To this date, the authors have not found any literature on augmentation of the anterior maxilla with iliac crest bone block grafts with emphasis not only on implant survival but also on soft tissue aesthetics with the PES -scale. The aim of this retrospective follow-up study was thus to assess the survival rate of dental implants, gained horizontal alveolar bone width and the aesthetic outcome of the treatment with fixed all ceramic restorations.

## Material and Methods

Ethical Approval. The study was approved by the Internal Review Board of the Division of Musculoskeletal Surgery, Helsinki University Central Hospital, Helsinki, Finland. Patient Consent. Not required.

Nine patients, 5 females and 4 males, were included in this study. The average age of the patients was 27 years [19-39]. The inclusion criterion was severe bone atrophy in the anterior maxilla caused by trauma, tooth extraction or congenitally missing teeth. Severe atrophy meaning alveolar width ranging from 1-4mm marginally or 3mm or less at any site. Indication for augmentation for two sites where the width was > 4mm was augmentation of adjacent implant site.

Experienced oral and maxillofacial surgeons performed all surgical procedures. At first all patients underwent an augmentation operation, where 4-5mm thick autogenous bone blocks were harvested from the anterior iliac crest and transplanted to the atrophied premaxilla under general anesthesia. The intraoral incision was in most of the patients made not through the keratinised mucosa on the ridge, but in the free mucosa in the buccal sulcus thus avoiding tension stress at the edges of the wound during the healing period. The dentoalveolar ridge was exposed through the horizontal sulcus incision by an undermining technique designed by one of the authors (P.S) and the corticocancellous bone blocks were shaped optimally and fixed subperiosteally on to the ridge with mini screws (1.5-2.0, DePuy Synthes , Switzerland) and covered with a resorbable collagen membrane (Bio-gideâ, Geistlich, Germany) in most of the cases ( Table 1). The wound was closed with resorbable sutures.

**Table 1:**
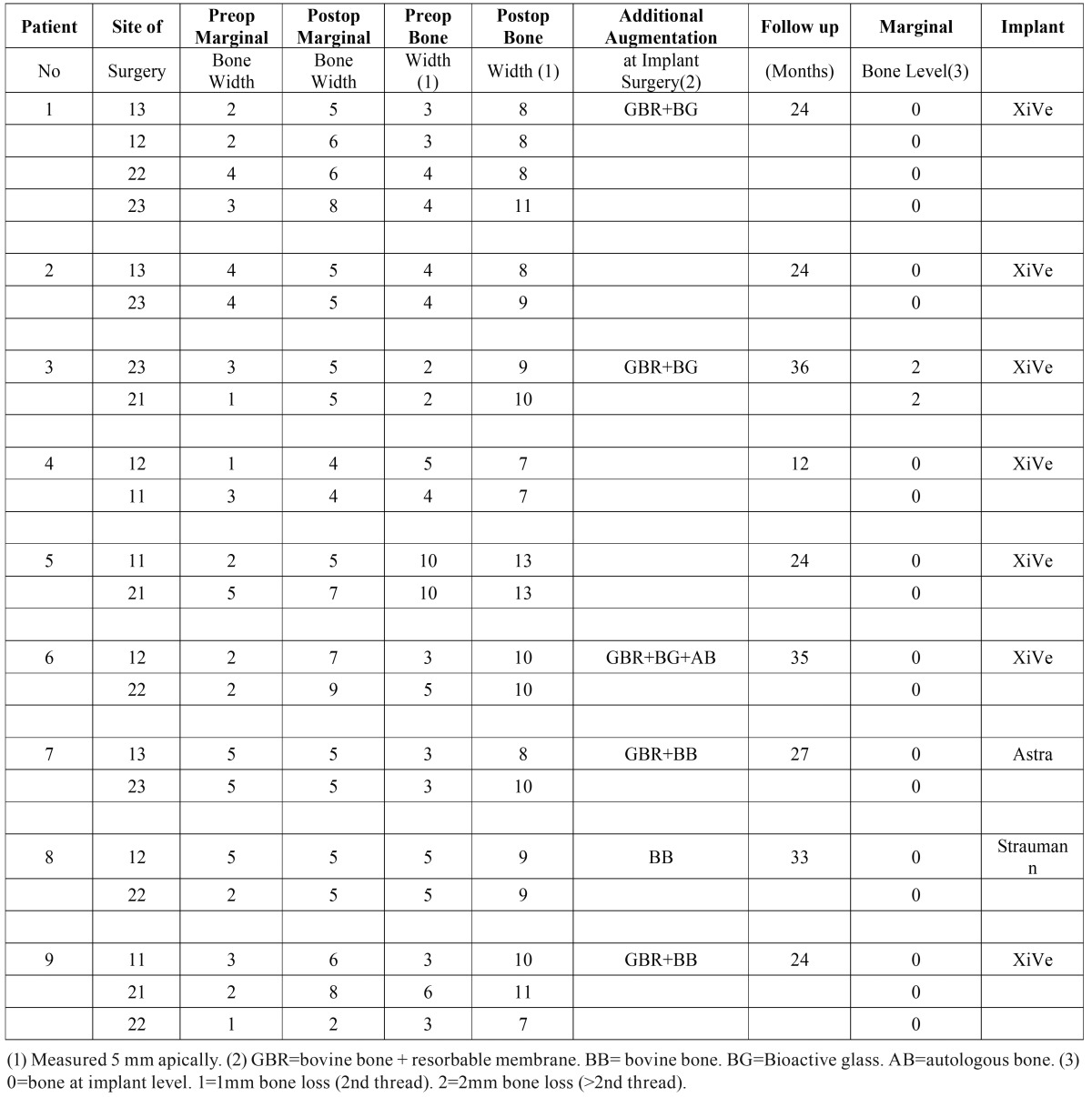
Results of augmentation with corticocancellous boneblocks from the iliac crest and survival rate of dental implants.

**Figure 1:**
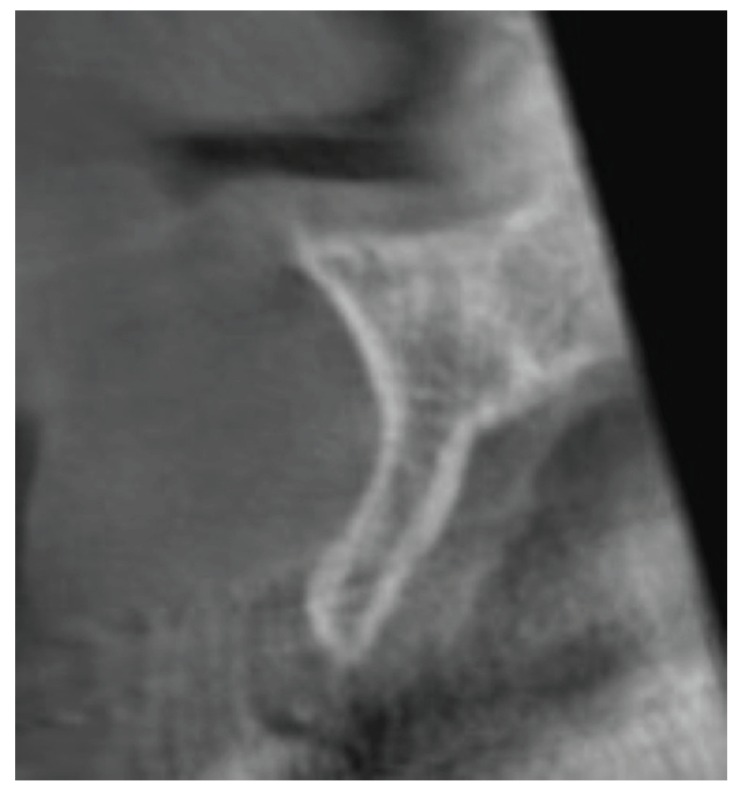
Preoperative sagittal view of the dentoalveolar crest reg 12 (Patient number 6).

**Figure 2:**
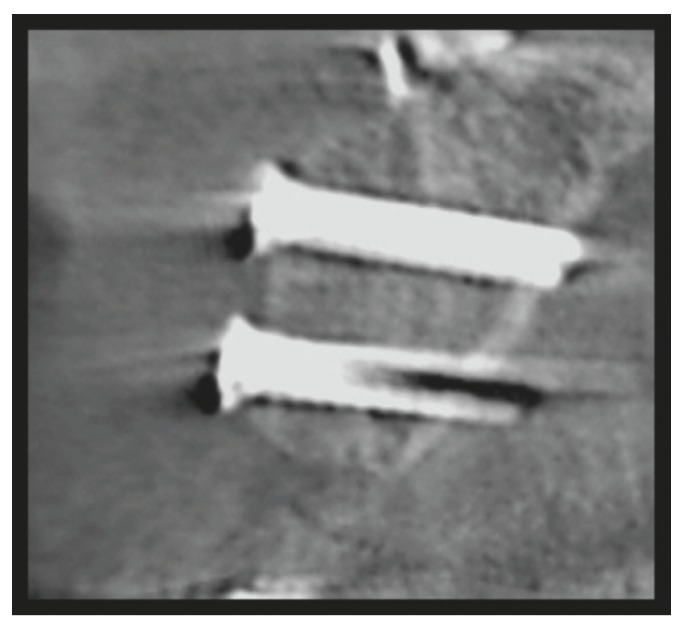
Postoperative sagittal view of the dentoalveolar crest reg 12 (Patient number 6).

Cone-beam computed tomography (CBCT) x-rays were taken before and 3-6 months after the augmentation surgery (Figs. 1,2). The thickness of the alveolar ridge was recorded pre- and postoperatively at the crest and 5 mm below the crest.

In the second stage, 3-6 months after the augmentation procedure, the application of dental implants was planned and performed according to the postoperative CBCT-scans. In some cases a secondary augmentation to improve the aesthetic outcome was performed at this stage using GBR or bone substitute without a collagen membrane (Bio-guideâ, Geistlich, Germany) ( Table 1). The bone substitutes used were bovine bone (Bio-ossâ, Geistlich, Germany) and bioactive glass granules (BonAliveâ, BonAlive Biomaterials Ltd, Turku, Finland) ( Table 1). However, non-resorbabale membranes were not used. All implant surgery was done as 2-stage.

During the healing period after ridge augmentation and after application of implants the patients had a temporary removable prosthesis shaped to obtain and maintain the right contour of the gingival margin and papillas. The removable denture was adjusted by adding acrylic in the basis area corresponding to the implant site and no labial support was allowed.

At the third stage, after 3-6 months osseointegration, the implants were exposed and loaded either with temporary or final fixed all ceramic restorations with custom made abutments by a prosthodontist.

**Figure 3:**
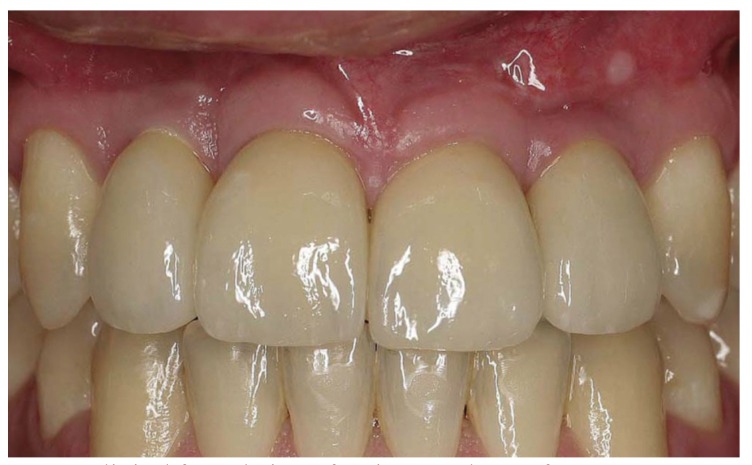
Clinical frontal view of patient number 6 after treatment. Implants placed in grafted bone reg 12, 22. Reg 12 the PES was 13, reg 22 the PES was 14.

The follow up period was counted from the time of loading of the implants. At the end of the follow up the patients were clinically and radiologically examined. The bone level at the implant sites was analyzed with dental x-ray images at the end of the follow up. According to the literature photographs can be reliably used for collection of PES data (17). In this study the aesthetic outcome of the rehabilitated area was evaluated by three different persons from close up high-resolution colour- calibrated photographs (Fig. 3) taken by the hospital´s photographer. The aesthetic outcome of the keratinised mucosa and soft tissue in the rehabilitated area was analysed using the PES-scale including 7 variables: 1) formation of the mesial and 2) distal papilla, 3) level of the gingival margin, 4) form of the gingival margin, 5) form of the alveolar process, 6) contour of the keratinised mucosa / soft tissue, and finally 7) the soft tissue colour invented by Furhauser *et al.* (13). The maximum aesthetic score per criteria being 2 indicating perfect aesthetics, 1 moderate change and 0 obvious change. The maximum PES thus being 14 per implant site analysed. The biotype was also registered ( Table 2).

**Table 2:**
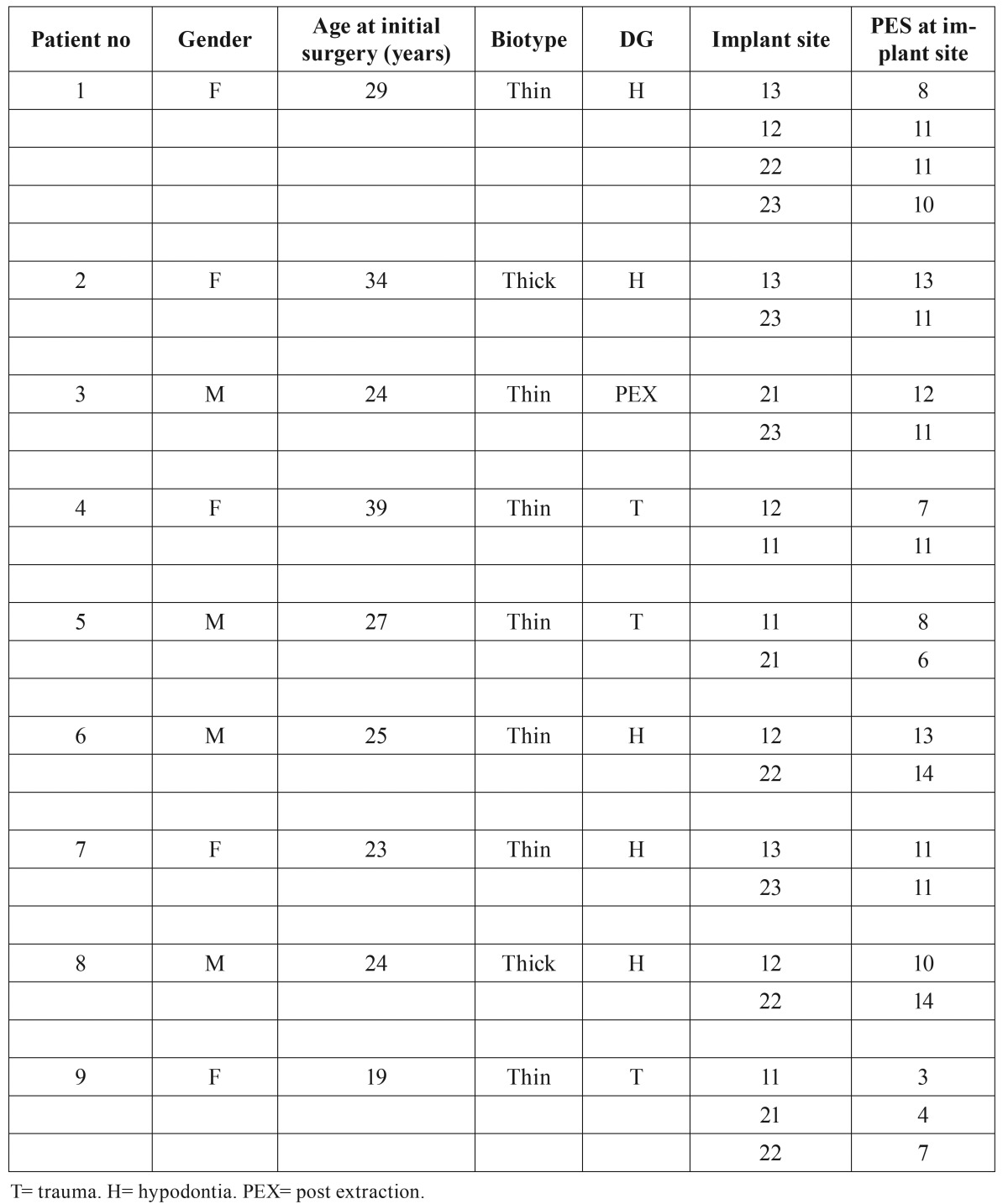
Biotype, Diagnosis and Pink Aesthetic Score.

## Results

The patients were followed up for an average of 27 months (12 - 36 months). The corticocancellous bone grafts osseointgrated successfully in all patients and the survival rate of the grafts was 100%. All nine patients had enough bone to accommodate dental implants and prosthetic rehabilitation with all ceramic restorations and custom made abutments was performed in all cases.

The changes in the amount of the horizontal dimension of the bony dentoalveolar ridge was measured marginally and 5 mm apically. The gained horizontal bone at the marginal level was in average of 2.7 mm and 5mm apically measured 5.0 mm. These results and the survival rate of the implants are shown in  Table 1.

We found that the shape of the temporary prosthesis played a major role on the formation of the soft tissue during the healing period. The adjusted prosthesis improved the formation of the maturating mesial and distal papillas as well as the shape of the gingival margin.

All 21 implants were clinically and radiographically stable after the average follow-up time of 27 months, counted from loading of the implants. The bone level was stable in 8 patients. In one patient crestal bone loss was below the second thread in both implant sites. This patient had severe atrophy of the dentoalveolar crest following tooth extraction and additionally he had a thin biotype. The survival rate of the implants was 100%.

The PES score was analysed from high resolution colour-calibrated photographs taken in average 27 months (12-36 months) after loading the implants and evaluated by three different persons. In table 2 the PES values for each patient’s implant site is presented. The mean PES rating for 21 implant sites was 9.8 out of 14 possible. In this study the PES was less than 8 at the site of 5 implants 

Differences could be seen in the PES according to the diagnosis and biotype. For patients with a thick biotype or a thin biotype it was accordingly 12 and 9,3.

The lowest PES rating was seen in trauma patients. The mean PES was 6.6 in patients with trauma, 11.4 in hypodontia patients and 11.5 in patients with previously extracted teeth.

Two of the patients had a thick biotype and 7 a thin biotype. All the patients with the PES being less than 7 were trauma patients with a thin biotype.

The skin scar at the anterior iliac crest varied from 2-4 cm in the patients. None of the patients had any problems with hypoesthesia or allodynia at the donor site at the end of the follow up, and all 9 patients healed uneventfully. No keloid scar formation was either seen in any of the patients.

## Discussion

Rehabilitation of the anterior maxilla with fixed restorations on implants demands not only a functional outcome but also good aesthetics to meet the patient´s needs and expectations. A patient with a low smile line has the highest prognosis to be satisfied with the treatment. Aesthetic risk factors are; high smile line, regular gingival margin of the adjacent teeth, thin biotype, resorbtion of the dentoalveolar ridge and triangular shape of adjacent teeth. When the dentoalveolar ridge is less than 3 mm wide in the aesthetic region todays treatment goal; excellent function and excellent aesthetics demanding sufficient bony reconstruction with anatomically shaped keratinized mucosa is a challenge for the surgeon-prosthodontist team.

In our retrospective study nine patients were treated with augmentation of the anterior part of the maxilla due to severe atrophy. 21 implants were placed into the augmented areas and the survival rate of the bone grafts and implants was 100%.

Dental implantation in the augmented anterior maxilla is a well-documented procedure and the survival rates are similar to other parts of jaws (18,19). However, studies focusing on long-term aesthetic results of treatment are scarce (20) and are usually cases with only singe-tooth implant restorations (21,22).

The morphology of the alveolar bone determines the contour of the gingiva around dental implants. It has been shown previously that there is a correlation between the size of the bone defect and the extent of the gingival recession affecting the aesthetic outcome (23). In the present study we had 6 cases where two to three adjacent teeth were replaced by implants after augmentation. In these cases the PES-scores were significantly lower with implant-implant interface being in average 7.4 compared to implant- tooth interface; being in average 11.6. However, according to the literature the threshold for clinical acceptability is ranging from PES ≥ 8 (15) and PES over 6 (16).

Despite the fact that the implants were placed into grafted bone, the bone level was very well maintained. Marginal bone resorption over the 2nd thread was seen only in one patient. This was a patient with a thin biotype. Even thus a thin biotype is considered to have a significant influence on marginal bone stability around implants increasing the risk of resorption (24), this phenomenon was seen only in one patient with a thin biotype. The PES was, however, 11-12 at the site of marginal bone loss.

The formation of the papilla is maybe the most important factor when measuring the aesthetic result of the treatment. With two or more adjacent implants, the formation of the papilla is more demanding to achieve than with a tooth next to the implant crown. In a study by Cosyn and co-workers 2013 (25) the embrasure and papilla heights between tooth-implant and implant-implant interfaces were measured. They found that between implants the papilla height is almost one mm shorter than with a tooth adjacent to the implant (4.1mm compared to 3.3mm).

We found that the aesthetic outcome was slightly better in patients with a thick biotype than in patients with a thin biotype. Our findings were thus in line with earlier findings, showing that a thin and narrow mucosa may lead to greater marginal recession, which is of significant importance in the aesthetic region (26).

Differences could be seen in the PES according to the diagnosis and biotype. Four implants were put into patients with thick biotype. Of these four implants the PES was > 8 at all of the implant sites. The corresponding rate in patients with a thin biotype was 17 implants, of which the PES < 8 in three patients at a total of five implant sites. These three patients with the PES < 8 were all trauma patients. The mean PES rating was 9.8. For patients with a thick biotype or a thin biotype it was accordingly 12 and 9.3. The lowest mean PES rating was seen in trauma patients; 6.6. Thus, patients with hypodontia or tooth extraction showed better aesthetic outcome than patients that had prior trauma in the rehabilitated area. Earlier surgery and scar formation is known to impair the healing process of both hard and soft tissue, which also could be seen in the present study. Most of the trauma patients also suffered from vertical deficiency of the alveolar process.

The PES was slightly better in areas were only single implants were put compared to multiple adjacent implants. This is correlating to the amount and nutrition of the bone between the implants as earlier shown (25), since two adjacent implants needs more “interdental space” than an implant and a tooth. The distance from the bone level to the approximal contact is critical for optimal papilla formation and the design of the implant crown and the crown of the adjacent teeth are of big importance (27). The height of the papillas is also more demanding to re-establish the larger the edentulous area is. The PES was >8 in all implants with adjacent teeth on both sides. These findings are in line with the literature showing that the re-establishment of soft tissue and papilla height is difficult and pontic may not perform better than adjacent implants (25).

We also found that the right shape of the temporary prosthesis played a major role on the formation of the soft tissue during the healing period improving the formation of the mesial and distal papillas as well as the shape of the gingival margin.

As a conclusion, we found that reconstruction of the atrophied anterior maxilla with corticocancellous bone blocks from the iliac crest is a safe procedure with a high survival rate for gained horizontal width and dental implants. Occurred trauma in the premaxilla often resulting in mucosal scar formation, and both horizontal and vertical bone loss combined with a thin biotype predicts a low PES.
